# Impact of inhalational anesthetics on postoperative cognitive function: Study protocol of a systematic review and meta-analysis

**DOI:** 10.1097/MD.0000000000009316

**Published:** 2018-01-05

**Authors:** Yi-Qing Zou, Xiao-Bao Li, Zhi-Xing Yang, Jing-Min Zhou, Yi-Nan Wu, Zhi-Hu Zhao, Xiang-Zhu Liu, Chang-Li Hu

**Affiliations:** a Department of Anesthesiology and Intensive Care Unit, the 476 Hospital of Fuzhou General Hospital; b College of Clinical Medicine of Fuzhou General Hospital, Fujian Medical University, Fujian Province, China.

**Keywords:** inhalational anesthetics, network meta-analysis, postoperative cognitive function, systematic review protocol

## Abstract

**Background:**

Conflict findings of the impact of inhalational anesthetics on postoperative cognitive function are reported. No systematic review has been performed to solve the problem. The aim of the study was to assess the effect of different inhalational anesthetics on postoperative cognitive function in a network meta-analysis.

**Methods:**

We will search MEDLINE, EMBASE, the Central Register of Controlled Trials in the Cochrane library, and CINAHL for randomized controlled trials or cohort studies assessing the short-term or long-term cognitive function of elderly patients (over 60 years) receiving major surgeries and inhalational anesthetics (desflurane, isoflurane, sevoflurane, halothane, and nitrous oxide) during surgery. Two reviewers will independently screen study eligibility, extract information from eligible studies, and appraise study quality. The impact of inhalational anesthetics will be assessed through: incidence of postoperative cognitive dysfunction at 1 week, 3 months, 1 year, and over 1 year after surgery; incidence of post-operative delirium; test of postoperative cognitive function.

**Results:**

The results of this systematic review and meta-analysis will be published in a peer-reviewed journal.

**Conclusion:**

To our knowledge, this systematic review will be the first to evaluate existing research on the incidence of postoperative cognitive function after inhalational anesthetics. Our study will assess the effect of different inhalational anesthetics on postoperative cognitive function.

**Ethics and dissemination:**

The review will be finished in December 2017, and the result will be published in a peer-reviewed journal or disseminated through conference posters or abstracts.

**Review registration number:**

CRD42017056675 (www.crd.york.ac.uk/PROSPERO).

## 1 Introduction

Postoperative cognitive dysfunction (POCD), a complication after surgery, is closely related to low quality of life in patients receiving major surgeries. POCD at 3 months after surgery indicates a 1.6 times higher rate in mortality; it also increases the risk of disability or voluntary early retire.^[1]^ In United Kingdom, 26.5% of patients develop POCD after receiving surgery for 1 week, and 9.9% of patients still have POCD after 3 months.^[2]^ Global prevalence of POCD is not reported, mainly because POCD is not clearly defined in published studies, and assessment methods are significantly heterogeneous in these studies.^[3]^

The risk of POCD is associated with increasing age, anesthesia, low education level, repeated surgeries, postoperative infections, and respiratory complications.^[2]^ General anesthesia is often mentioned as a possible cause of POCD, since it works through the neurological system. Bedford retrospectively reviewed 1193 elderly patients who had received general anesthesia during surgeries; he found 10% of them had POCD 5 years after surgery.^[4]^ Two studies compared general anesthesia with regional anesthesia in causing POCD, and the results are conflict.^[5,6]^ A systematic reviews and meta-analysis showed that general anesthesia induced higher incidence of POCD than regional anesthesia,^[7]^ but some scholars argue that it may be the consequence of receiving major surgeries or having severe inflammatory response in the brain.^[8,9]^

Using inhalational anesthetics (IAs) is the mainstay of general anesthesia; IAs are used as maintenance of anesthesia after intravenous anesthesia. Since they pass readily into the brain, IAs are usually recognized as the most important cause of POCD. Concentrations of halothane and isoflurane caused aggregation of amyloid peptides in cell cultures, indicating that they brought cytotoxicity to the brain^[10]^; sevoflurane also showed the same cytotoxic effect.^[11]^ However, some studies showed that IAs had a protective effect on postoperative cognitive function. One study found that 2% isoflurane was associated with better postoperative performance than 1% isoflurane in postoperative cognitive function testing,^[12]^ indicating the depth of anesthesia played an important role postoperative cognitive function. Additionally, patients receiving propofol-based anesthesia are inclined to higher incidence of POCD compared to those receiving sevoflurane^[13]^ or desflurane.^[14]^ The above 3 studies showed that IAs had a protective effect on postoperative cognitive function. Inconsistent findings of the impact of IAs on post-operative cognitive function raise questions: (1) Do IAs have protective effect on post-operative cognitive function? (2) If so, which IA has the best protective effect? Several studies have been conducted to answer the questions,^[11,13–16]^ but conflict findings hinder drawing a firm conclusion, so we will conduct a systematic review and network meta-analysis trying to answer the questions.

## 2 Methods and analysis

**Figure 1:**
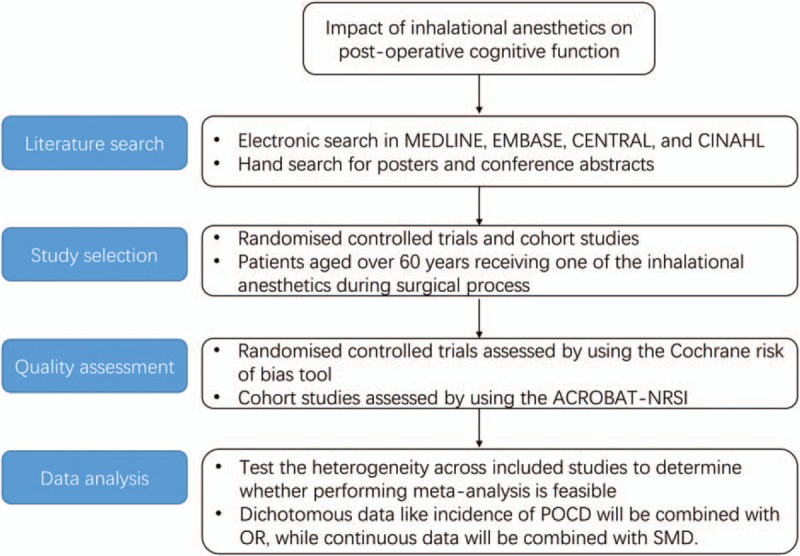
Flow chart. The ACROBAT-NRSI is a Cochrane risk of bias assessment tool for nonrandomized studies of interventions. OR = odds ratio, POCD = postoperative cognitive dysfunction, SMD = standardized mean difference.

The protocol of the systematic review and network meta-analysis is reported in accordance with PRISMA-P guidelines.^[17]^Figure 1 shows the flowchart of the review.

### 2.1 Selection of studies

We will include studies assessing the effect of IAs on postoperative cognitive function of patients receiving major surgeries, defined as surgical procedures during which patients are under general anesthesia.

### 2.2 Study design

We will include randomized controlled trials or cohort studies with a follow-up period longer than 3 months. Trials with cross-over design will be excluded, since the test–retest reliability is questioned in evaluation of postoperative cognitive function.

### 2.3 Patients

We will include patients with age over 60 years and those receiving desflurane, isoflurane, sevoflurane, halothane, or nitrous oxide in general anesthesia. The type of surgery is not limited, but it will be classified as cardiac or noncardiac surgery, since cardiac surgeries, especially cardiopulmonary bypass surgeries (CPB), tend to induce worse POCD than non-cardiac surgeries.^[9]^ We will include studies using mini-mental state examination (MMSE) as a screening tool to exclude preoperative cognitive impairment. Studies including patients with established cognitive impairment before surgery (Alzheimer's disease, vascular dementia, Parkinson-dementia, frontotemporal dementia) will be excluded.

### 2.4 Outcome measurements

The primary outcome will be cognitive failure questionnaire (CFQ), consisting of 25 questions about minor mistakes that everyone possibly makes in daily life. Each question has 5 options recording the frequency of making mistakes, from very often (4 points) to never (0 point). We will sum up the points of the 25 questions generating a total score; a higher total score indicates a worse cognitive state. The CFQ has been used in several studies investigating postoperative cognitive dysfunction,^[2,18–20]^ and it is acknowledged for its reliability and validity in assessing postoperative cognitive function.

Secondary outcomes include 4 neuropsychological tests. The first, visual verbal learning test (VVLT) will be used to assess memory defect. We will count cumulative number of words that patients recall in 3 tests and the number of words at delayed recall. The second test will be a concept shifting test (CST); it measures concept shifting and executive functioning.^[21]^ We will calculate the time to finish the test and the number of errors in the part C of the CST. The third test will be the Stroop Color Word Test (SCWT)^[22]^; it is a 5-minute test for assessing cognitive processing and providing diagnostic information on cognitive function. We will assess the time to finish the test and error scores from the third part of the SCWT. The fourth test will be the Letter Digit Coding Test (LDCT),^[23]^ also known as digit symbol substitution test. LDCT consists of digit-symbol pairs followed by a list of digits. Under each digit the patient should write down the corresponding symbol or letter as soon as possible. The number of correct answers for the LDCT test will be recorded.

Since mood disturbance is related to POCD,^[24]^ we will also assess the mood status of patients by using the Geriatric Depression Scale (GDS)^[25]^ or Zung self-rating depression scale (SDS)^[26]^ or Zung's self-rating anxiety scale (SAS),^[27]^ all of which are frequently used as screening tools for assessing mood disturbance. Two reviewers (ZXY and JMZ) will independently screen studies according to the aforementioned criteria, and agreement between the 2 reviewers will be assessed with kappa statistic.^[28]^

### 2.5 Search strategy

We will search the following electronic databases: MEDLINE (via OVID), EMBASE (via www.embase.com), the Central Register of Controlled Trials (CENTRAL) hosted by the Cochrane library, and CINAHL. We have developed a search strategy with combination of the following MeSH terms or keywords: postoperative cognitive function, cognitive dysfunction after surgery, general anesthesia, inhalational anesthetic, inhaled anesthesia, and so on. Table 1 shows the full search strategy.

**Table 1:**
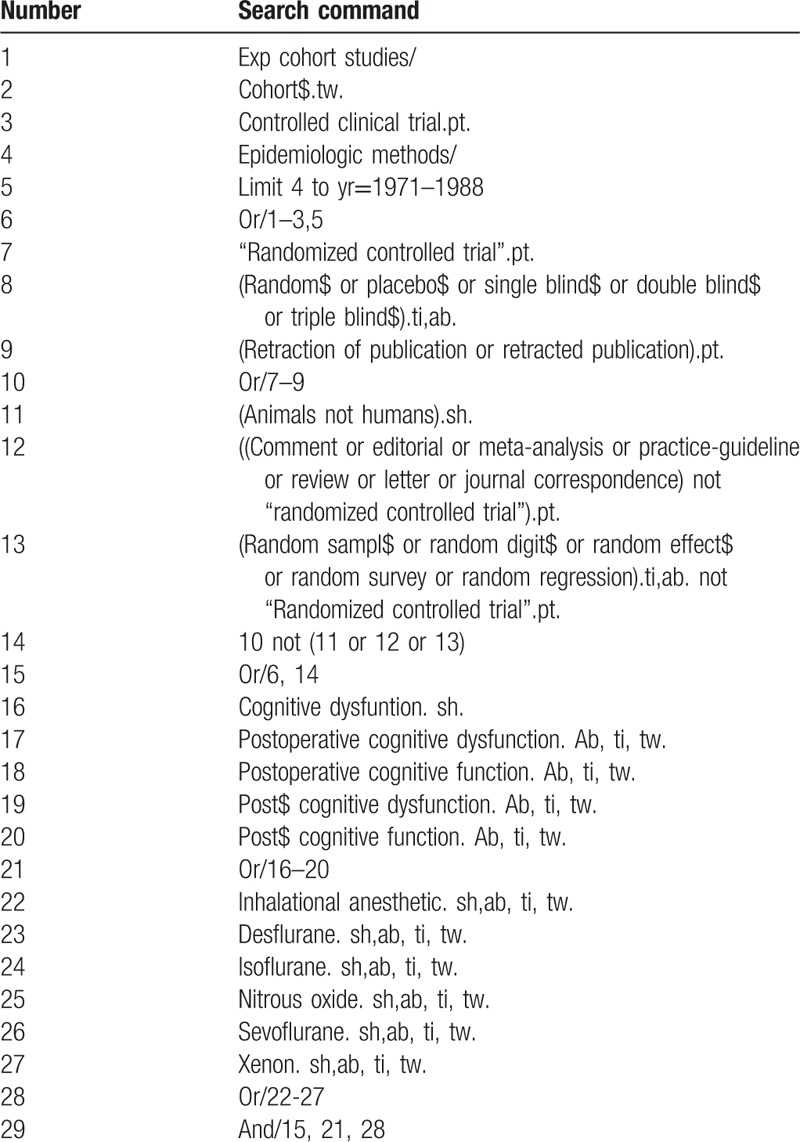
Search strategy via Ovid MEDLINE.

We will also search the following website to find unpublished studies: www.clinicaltrials.gov, http://www.fda.gov/ (for searching data of post-marketing adverse events of inhalational anesthetics), http://www.iars.org/ (the website of International Anesthesia Research Society), and http://www.stahq.org/ (the website of Society For Technology in Anesthesia). We will also contact pharmacy companies manufacturing inhalational anesthetics for unpublished data. Additionally, we will check systematic reviews assessing the impact of surgery and anesthesia on cognitive function for trials eligible for our review.

### 2.6 Data extraction

Standardized forms will be developed for data extraction. We will record the source of the included studies about study ID, report ID, citation, and contact details. Reasons for eligibility and exclusion will also be recorded. This review includes both experimental and observational studies, so we will extract the information of study design, total study duration, and settings. Information of participants is the key to accurately evaluate the true impact of inhalational anesthetics. We will record the total number of participants, age, sex, diabetes (yes/no/unclear), hypertension (yes/no/unclear), and family history of dementia or mild cognitive impairment (yes/no/unclear), since patients with age over 65 years showed cognitive impairment when they accompanied with diabetes^[29]^ and hypertension.^[30,31]^ Information about the surgeries performed and the analgesic methods used will be extracted. The surgeries will be recorded as cardiac surgeries and noncardiac surgeries, and noncardiac surgery will be further described in detail as open surgery, laparoscopic surgery, microsurgery, and other major surgeries including organ transplant surgery, or some types of emergency surgery. The use of inhalational anesthetics will be recorded as introduction of anesthetic, maintenance of anesthetic after introduction, or both of them (the exact name of an IA will be recorded). The length of follow-up period, outcome measurements (incidence of POCD, preoperative or postoperative cognitive function, or scales for assessing mood disturbance before or after surgery), and results of outcome measurements will be recorded for further analysis.

### 2.7 Risk of bias assessment

The quality of included randomized controlled trials will be assessed by using the risk of bias tool provided by the Cochrane library. We will assess randomized controlled trials in 6 aspects: random sequence generation, allocation concealment, blinding of participants and personnel, blinding of outcome assessor, incomplete outcome data, selective reporting, and other source of bias. Assessment results of the 6 aspect will be summarized into a risk-of-bias figure, generating an overall impression of the quality.

The quality of nonrandomized studies will be assessed by using the ACROBAT-NRSI,^[32]^ which includes 7 chronologically arranged bias domains (preintervention, at intervention, and postintervention). Two domains of bias are assessed in preintervention (bias due to confounding and selection of participants); one is assessed at an intervention stage (bias in measurement of interventions); four domains are evaluated at the postintervention stage (bias due to departures from intended interventions, missing data, measurement of outcomes, and selection of the reported results). Each of the 7 domains will be classified as low, moderate, serious or critical risk of bias, and the classification of the 7 domains will be presented in a table to show overall quality of included studies.

### 2.8 Data synthesis

Before performing data synthesis, we will check if it is possible to perform meta-analysis. Both clinical heterogeneity and statistical heterogeneity will be examined. For clinical heterogeneity, we will present the baseline characteristics (age, gender, accompanied diseases, concomitant drugs of participants) in each study, the settings where the included studies are carried out, and experience of surgeons (presented as years of practice), to check if these parameters are homogeneous in included trials. For statistical heterogeneity, we will first calculate the effect size of a certain IA on incidence of POCD or other measurements on postoperative cognitive function, and second we will compare the effect sizes across different studies to check if they are consistent. Consistency of the effect sizes will be measured by the *I*^2^ statistics, and an *I*^2^>50% indicates existence of significant heterogeneity. If significant clinical heterogeneity or statistical heterogeneity exists, we will first try to find out the source of heterogeneity by meta-regression (using the Metafor package in R 3.3.1), and second we will perform subgroup analysis. Narrative review will be performed when heterogeneity still exists after meta-regression and subgroup analysis.

If it is possible to perform meta-analysis, we will calculate the effect size of each IA in each included trials, with relative risk (RR) for assessing incidence of POCD and standardized mean difference (SMD) for scales measuring postoperative cognitive function. We will first combine the effect sizes calculated from studies with head-to-head comparisons (e.g., several studies have assessed the impact of sevoflurane on postoperative cognitive function by comparing it directly to isoflurane, then we will combine the SMD of each study). When different titrates of a certain IA are compared in a study, we will separately calculate the effect size of each titrate. We will also calculate the effect size from indirect comparisons (e.g., supposed that we have studies compared sevoflurane with isoflurane and studies compared isoflurane with xeon, we could simulate an indirect comparison between sevoflurane and xeon). We will examine the consistency between direct and indirect comparisons before we perform network meta-analysis. We will adopt a Bayesian method to perform the network meta-analysis by using WINBUGS 1.4.

Several sensitivity analyses will be performed. First, we will exclude studies that are classified as the high risk of bias by the Cochrane risk of bias tool or the ACROBAT-NRSI, to check if the quality of the studies significantly modifies the results. Second, we will exclude studies in which patients receive cardiac surgeries, to find out whether cardiac surgery increases the risk of POCD.

## 3 Discussion

This is the first systematic review synthesizing the direct and indirect evidence of the impact of inhalational anesthetics on postoperative cognitive function, and it will be the first network meta-analysis conducted to determine the optimal regimen for using IAs in major surgeries. This protocol is designed according to the PRISMA-P guideline; the results of the network meta-analysis may help the patients and their physicians select the most suitable IAs before surgery.

### 3.1 Ethics and dissemination

This is a systematic review of published studies, and no ethical approval is needed. Results of the review will be submitted to a peer-reviewed journal for publication. The results will also be demonstrated through conference abstracts or posters.

Strengths and limitations of this study

- This will be the first network meta-analysis to evaluate the effect of different inhalational anesthetics on postoperative cognitive function.
- Using a combination of a comprehensive search strategy and the method of umbrella systematic review will ensure a thorough literature search.
- There will be heterogeneity in eligible studies, especially in outcome assessments for cognitive function.
